# Data on characterization, model, and adsorption rate of banana peel activated carbon (*Musa Acuminata*) for adsorbents of various heavy metals (Mn, Pb, Zn, Fe)

**DOI:** 10.1016/j.dib.2021.107611

**Published:** 2021-11-20

**Authors:** Khairiah Khairiah, Erna Frida, Kerista Sebayang, Perdinan Sinuhaji, Syahrul Humaidi

**Affiliations:** a Department of Physics, FMIPA, Universitas Sumatera Utara, Jl. Bioteknologi I Kampus USU Medan, Indonesia; b Universitas Muslim Nusantara Al Washliyah, Jl. Garu II A No. 93, Medan Amplas, Kota Medan, Indonesia

**Keywords:** Banana peel activated carbon, Adsorption, Adsorbent, Heavy metal (Mn, Pb, Zn, Fe), Polluted water

## Abstract

This research contains data on the adsorption test and characterization of banana peel activated carbon as an adsorbent for water contaminated with various heavy metals. Banana peel is a common post-harvest waste, making it easy to obtain. Atomic Absorption Spectroscopy (AAS) is used to investigate the various heavy metal content in polluted water that are harmful to health, such as Mn, Fe, Zn, and Pb in concentrations of 1.351; 1.210; 17.403; and 0.210 mg/L, respectively. These metals exceed the standard limits for clean water quality that are suitable for sanitation hygiene purposes. Banana peel activated carbon samples were tested in polluted water and re-examined using AAS. The parameters used to calculate the percent adsorption of the four heavy metals were variations in adsorbent mass (0.5; 1.0; 1.5; 2.0 g), stirring speed (50; 100; 150; 200; 250 rpm), pH (4; 5; 6; 7; 8; and contact time (30; 60; 90; 120; 150 min). Scanning Electron Microscopy/X-Ray Spectroscopy Energy Dispersion (SEM/EDS) and X-Ray Diffraction (XRD) were used to characterize and determine the morphology, material content, and crystal structure formed by the samples. Brunaurer, Emmet, and Teller/Barret, Joyner, and Halenda (BET/BJH) were used to investigate the surface area, pore size, and isotherm type. This dataset is publicly available for researchers to optimize the potential of banana peel activated carbon as an adsorbent of heavy metals (Mn, Pb, Zn, Fe) in the industrial sector.

## Specifications Table

**Table:** 

Subject	Materials Science
Specific subject area	Adsorption
Type of data	Table Graph Figure
How data were acquired	• % Adsorption data were obtained by measurement using AAS. • The characterization data for the crystal structure were measured using an XRD. • Morphological data and elemental content were measured using SEM-EDS. • Data on the surface area, pore size, and isotherm type were measured using BET-BJH
Data format	Raw and Processed
Parameters for data collection	Banana peel activated carbon adsorbent to adsorb heavy metals, such as Mn, Pb, Zn, and Fe. Parameters such as initial and final concentrations, % adsorption, surface area, pore size, and crystal size were collected.
Description of data collection	The banana peel activated carbon was characterized using AAS, XRD, SEM-EDS, and BET-BJH
Data source location	Desa Marindal II, Sumatera Utara, Indonesia
Data accessibility	Mendeley Data, V1, DOI: 10.17632/mcjwgpp6zg.1 https://data.mendeley.com/datasets/mcjwgpp6zg/1
Related research article	J. H. Khairiah, “Potential Banana Husk Waste (Musa Paradisiaca) For An Adsorbent,” *Int. J. Sci. Technol. Res.*, vol. 9, no. 3, pp. 1601–1604, 2020. http://www.ijstr.org/paper-references.php?ref=IJSTR-0320-31393

## Value of the Data

• The data can be used to predict the behavior of the banana peel activated carbon adsorption process on four heavy metals simultaneously (Mn, Pb, Zn, and Fe).
• The data can help the industry design adsorption columns that use banana peel activated carbon as a medium to remediate water contaminated with heavy metals (Mn, Pb, Zn, and Fe).
• Scientists can use the data to advance research by predicting and modeling adsorption capacity and interaction mechanisms in removing heavy metals like Mn, Pb, Zn, and Fe using banana peel activated carbon adsorbents.

## 1 Data Description

### 1.1 AAS analysis

The mixture of four different concentrations of metals (1.351; 1.210; 17.403; and 0.210 mg/L) have a total of 50 mL per sample. The sample was detected through AAS analysis tested on 0.5; 1.0; 1.5; and 2.0 g of banana peel activated carbon with varied stirring speed (rpm), pH, and contact time (min) at a constant temperature of 473 K. Tabel 1.1 shows optimum adsorption percentage (%) on a given mass of 2.0 g with a contact time, stirring speed, and pH of 120 min, 200 rpm, and 7 at 99.27%. The raw and processed data of adsorption percentage, with Langmuir and adsorption rates of pseudo-first and second orders, are presented in the Mendeley Data repository of ``Data Adsorption Banana.xlsx.''

### 1.2 SEM-EDS analysis

Characterization of SEM-EDS was carried out on sample results with a mass sample of 2 g, pH 7, 200 stirring speed, and contact time of 120 min as according to the data tabulated in Table 1.1.

**Table 1.1:** % Adsorption of heavy metals tested at various pH, stirring speed, and contact time at 473 K.

No	Materials	Heavy Metals	Mass (g)	pH	Stirring speed (rpm)	Contact Time (min)	Adsorption (%)	Temperature (K)
1	Banana Peels-Activated Carbon	Mn Fe Pb Zn	0.5	4	50	30	25.44	473
				5	100	60	38.57	
				6	150	90	47.85	
				7	200	120	64.89	
				8	250	150	58.91	

2		Mn Fe Pb Zn	1.0	4	50	30	24.65	
				5	100	60	38.55	
				6	150	90	49.82	
				7	200	120	74.89	
				8	250	150	56.93	

3		Mn Fe Pb Zn	1.5	4	50	30	35.69	
				5	100	60	47.54	
				6	150	90	56.90	
				7	200	120	75.83	
				8	250	150	68.78	

4		Mn Fe Pb Zn	2.0	4	50	30	52.14	
				5	100	60	68.87	
				6	150	90	80.61	
				7	200	120	99.27	
				8	250	150	87.83	

**Fig. 1.1:**
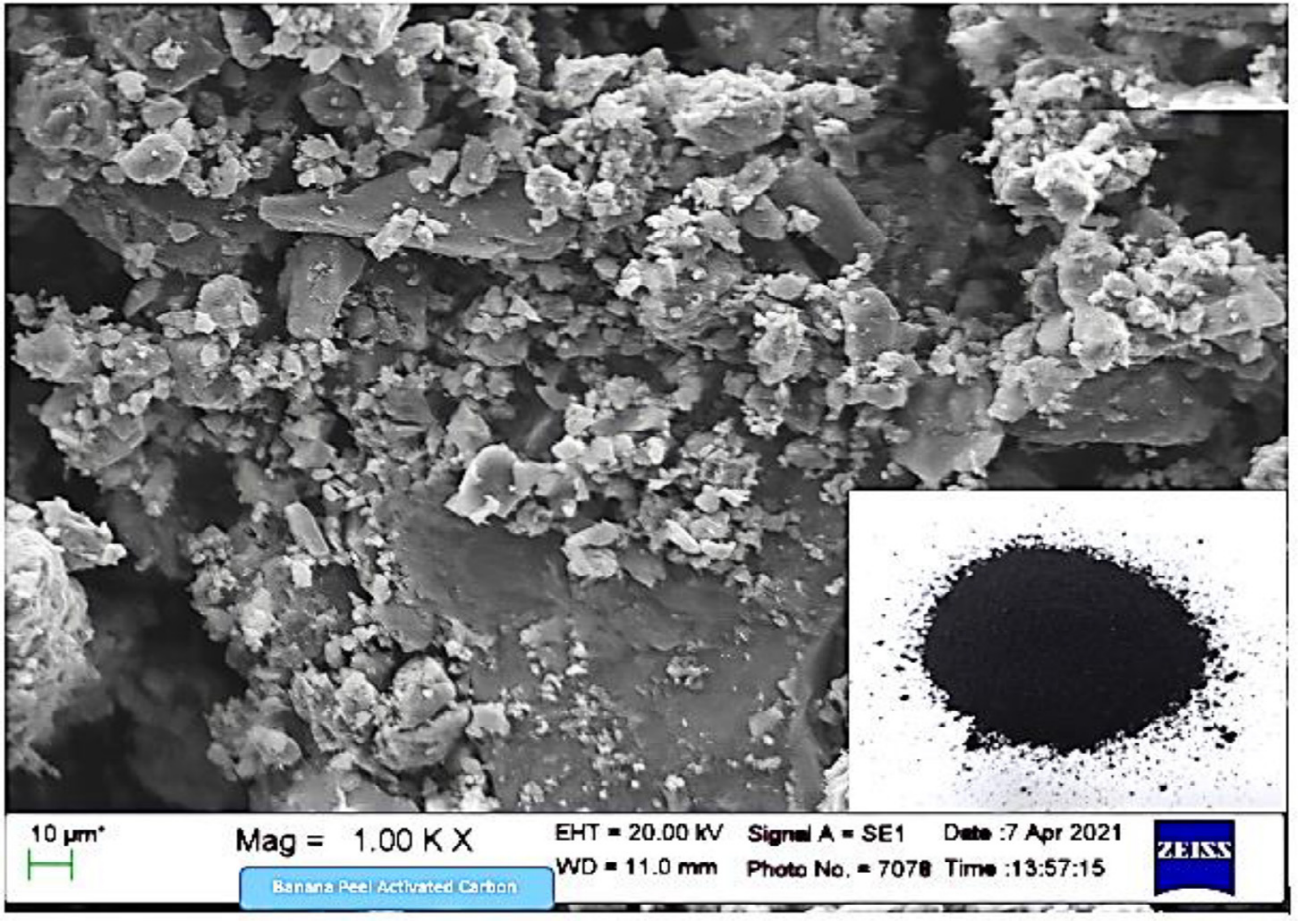
SEM image of banana peel activated carbon with 99.27% adsorption at a magnification of 1.00Kx.

**Fig. 1.2:**
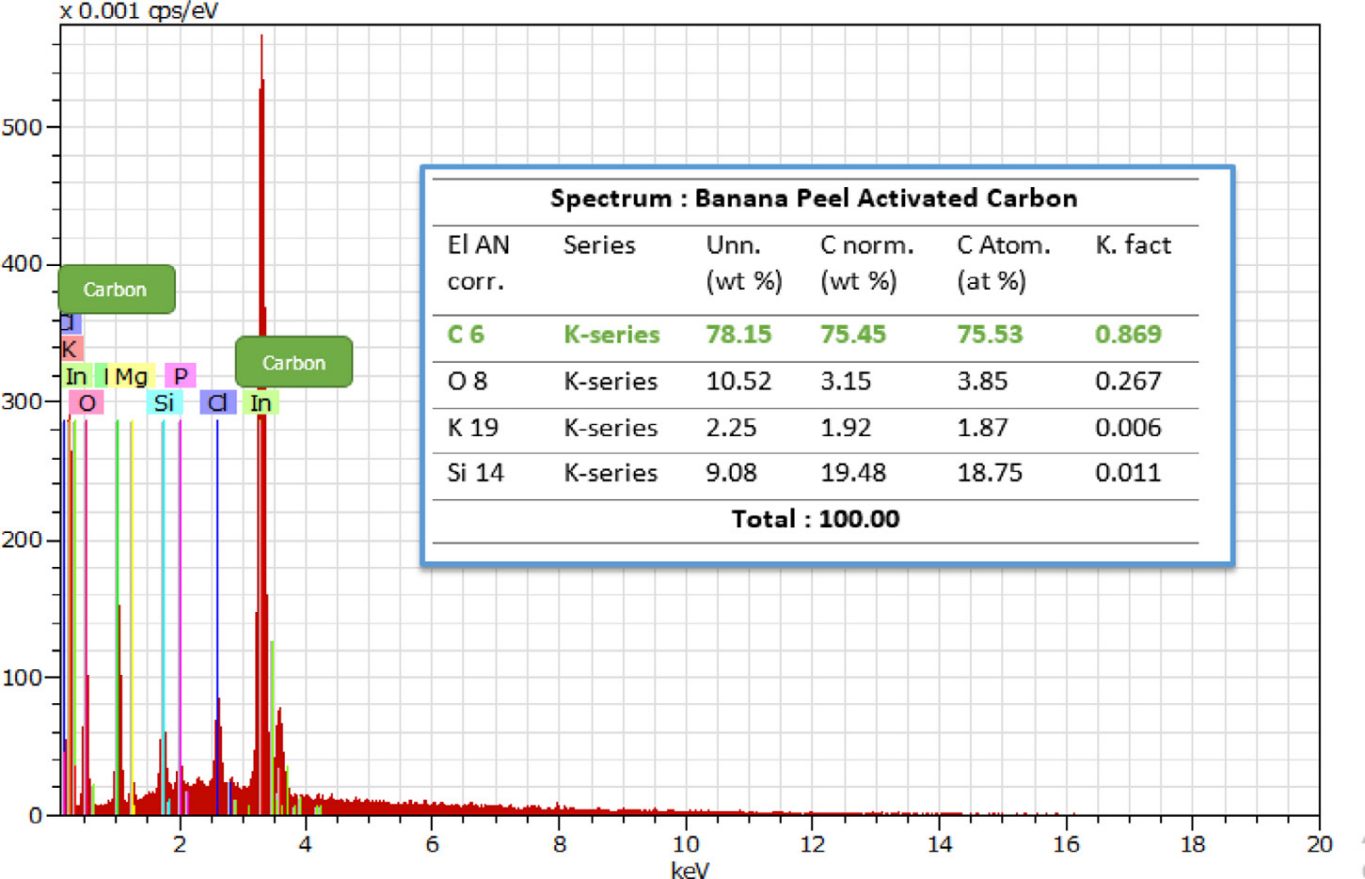
EDS Graph of banana peel activated carbon result for the adsorption of 99.27%.

The SEM-EDS sample was presented in the Mendeley Data repository "SEM_Banana Peel Activated Carbon 1 KX.tif". Fig. 1.1 shows the surface morphology of banana peel activated carbon with the highest adsorption at 99.27%, an irregular, amorphous, and agglomerated structure. Furthermore, the EDS characterization data in Fig. 1.2 shows that the activated carbon content of banana peel at 78.15% was elemental.

### 1.3 XRD analysis

**Fig. 1.3:**
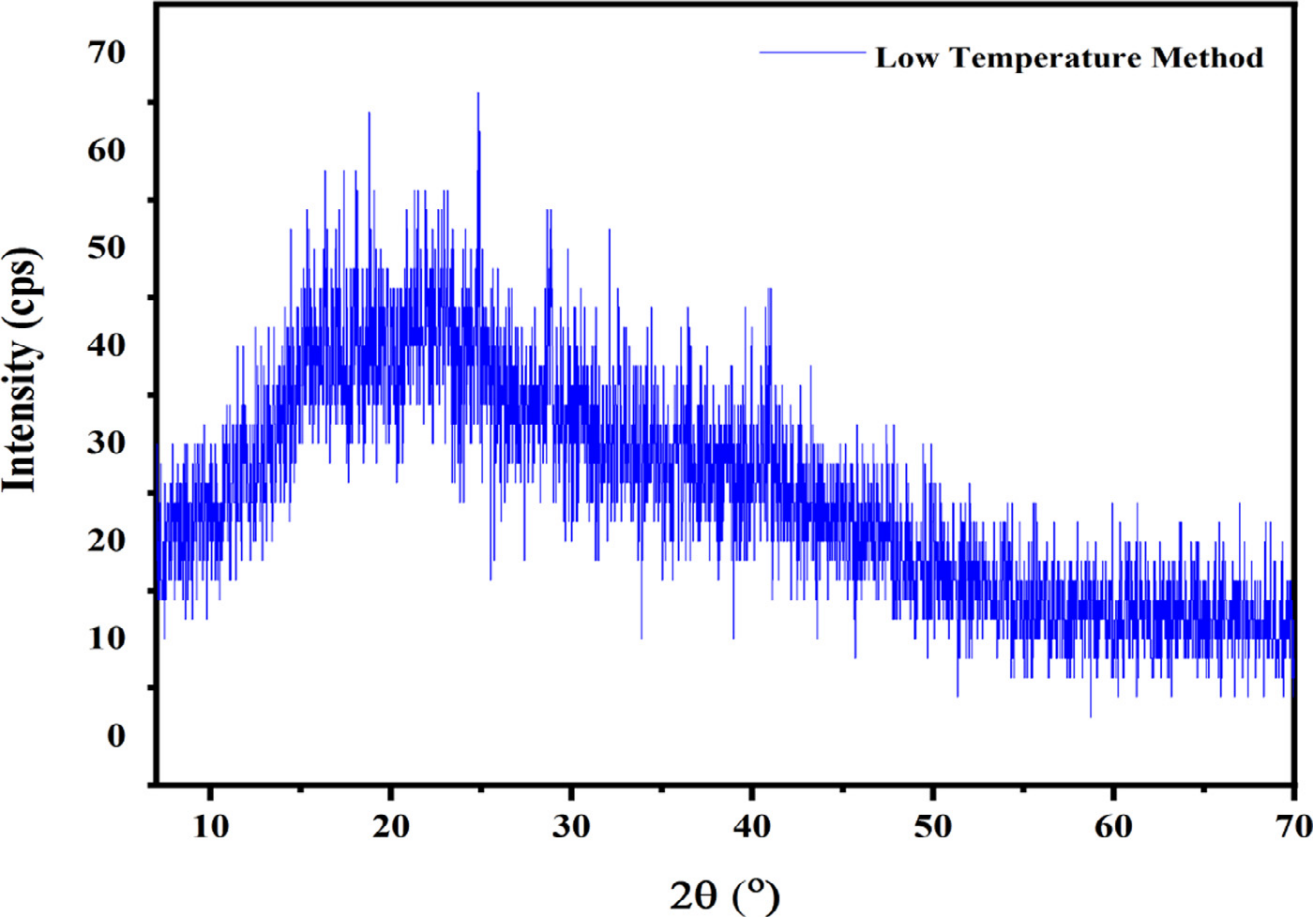
XRD Graph of banana peel activated carbon of adsorption 99.27%.

XRD characterization data were plotted using software (OriginLab), as shown in Fig. 1.3. The data recorded for 99.27% adsorption showed that the powder was amorphous with three strong peaks. Fig. 1.2 shows the XRD diffraction pattern of banana peel activated carbon with the examined samples consisting of the optimum % adsorption value. The sample shows its peak at 32.6^o^, 28.8^o^, and 28.7^o^, which corresponds to the data recorded in Table 1.2. The crystal sizes measured for each peak were 27.4, 30.9, and 28.7 nm. The XRD sample results were displayed in the Mendeley Data repository ``XRD_Banana.xls,'' ``XRD_Basic Process.pdf,'' and ``XRD RAW.pdf.''

**Table 1.2:** Calculation of the crystal size of banana peel activated carbon using the Scherer Equation.

K	A	K* λ	Center 2 α	Intensity	FWMH	Crystal size
0.9	1.54	1.386	32.6	**20**	0.0793	27.4 nm
0.9	1.54	1.386	28.8	**17**	0.1367	30.9 nm
0.9	1.54	1.386	28.7	**15**	0.0787	28.7 nm

### 1.4 BET-BJH analysis

**Fig. 1.4:**
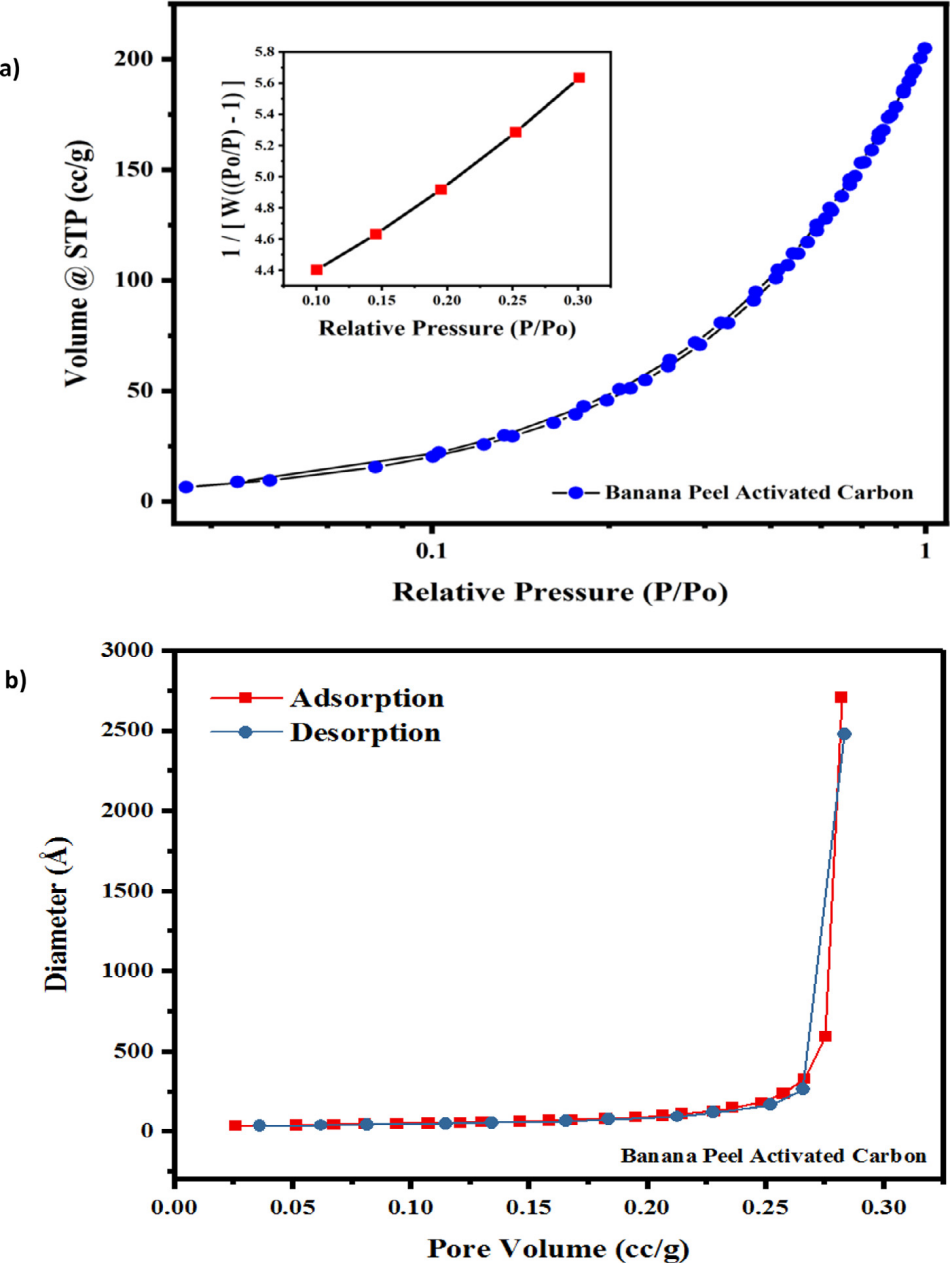
**(a).** The isothermic plot depicts the relationship between relative pressure and volume@STP on a 2 g mass of banana peel activated carbon; (**b).** The plot of the pore relationship and diameter of the adsorption and desorption processes on **the mass** activated carbon of banana peel.

The average pore size and surface area of ​​36.023 nm and 179.668 m^2^/g were obtained from the BET-BJH characterization. Fig. 1.4a shows a graph of the description BET-BJH with the relationship between relative pressure and standard temperature and pressure (STP) volume. Fig. 1.4b shows a graph of adsorption/desorption. The figure shows an increase in nitrogen gas at P/Po, which indicates an interaction between the adsorbent and the adsorbate in the International Union of Pure and Applied Chemistry (IUPAC) adsorption isotherm, including type III classification. The BET-BJH characterization data were displayed in the Mendeley Data repository under the names ``BJH Adsorption.txt, BJH Desorption.txt, Isotherm.txt, and Multipoint BET.txt.''

**Fig. 2.1:**
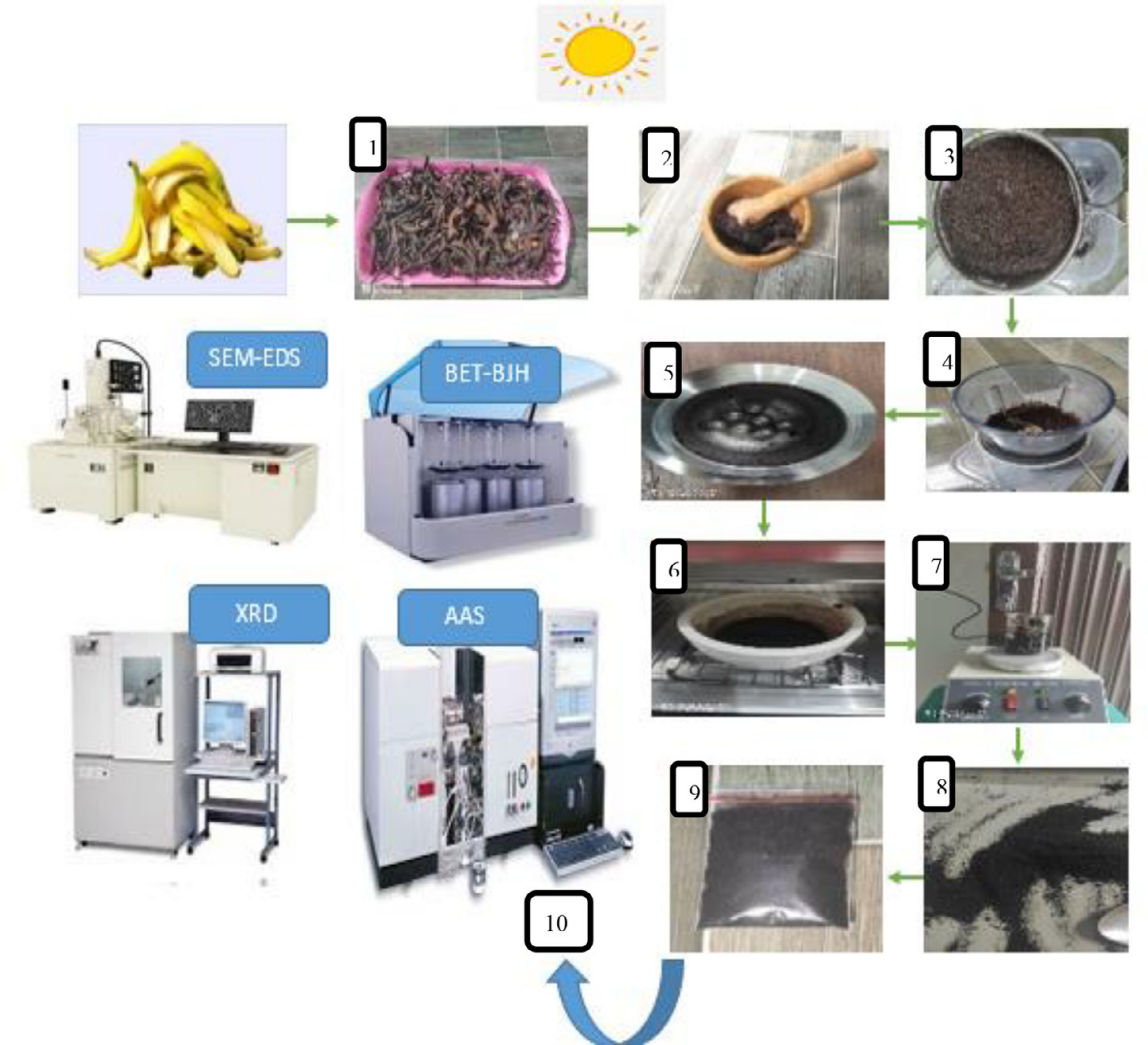
Preparation Flow of banana peel activated carbon.

## 2 Experimental Design, Materials and Methods

### 2.1 Materials

This research was conducted at the Laboratory of Material Physics, Universitas Muslim Nusantara Alwashliyah for four months. The polluted water was taken from one of the water sources in Desa Marindal II, Sumatera Utara, Indonesia, contaminated by four heavy metals. Namely, Mn, Fe, Zn, and Pb with initial concentrations of 1.351 mg/L, 1.210 mg/L, 17.403 mg/L, and 0.210 mg/L, respectively, thereby exceeding the standard limits for sanitation hygiene purposes. Only four types of materials were used in this study, namely banana peels, purified water (Aquades), hydrochloric acid (HCl), and sodium hydroxide (NaOH). The banana peels obtained approximately 3 kg of post-harvest waste, cleaned with Aquades to remove dirt on the inner surface. The HCl solution with a concentration of 25% was used chemically to activate the banana peels in maximizing the adsorption rate of heavy metals in polluted water. Meanwhile, the NaOH solution was used for the adjustment of pH variations in the observation tests.

### 2.2 Preparation

Firstly, the samples of banana peels were cleaned with distilled water (Aquades) and were further dried to reduce moisture content. The samples were pounded with a mortar and pestle before sieving and then blended again to achieve a fine powder. After that, the banana peel powder was inserted into High Energy Milling (HEM) for 12 h with 30 min breaks for each 90 min cycle [1]. Furthermore, the sample was carbonized at a temperature of 473 K for 24 h, and the substance was activated chemically using a diluted 25% concentration of HCl solution. Furthermore, the banana peel activated carbon was stirred for 2 h and then put into the oven at 80°C for 4 h. [2]. The preparation steps are illustrated in Fig. 2.1.

### 2.3 Adsorption procedure

The adsorbent test was carried out by inserting activated carbon from banana peels into 50 mL of heavy metal polluted water at initial concentrations (1,351; 1,210; 17,403; and 0,210 mg/L), adsorbent mass (0.5; 1.0; 1.5; 2.0 g), stirring speed (50; 100; 150; 200; 250 rpm), pH (4;5;6;7;8) with addition of 0.1 M NaOH solution and contact time (30;60;90;120;150 min), respectively. The obtained substance was then filtered with filter paper (brand: Whatman filter paper) to analyze the levels of adsorption of heavy metals by activated carbon of banana peels using AAS analysis. % Adsorption was determined as shown in the following equation.

(1) $$\%Adsorption=\frac{C_{o}-C_{e}}{C_{o}}\times \;100\%$$

With $c_{o}$: Initial Concentration (mg/l)

$c_{e}$: Final Concentration (mg/l)

### 2.4 Characterization

The study was carried out to analyze the surface morphology of the carbon material using SEM-EDS and its crystal structure using XRD. The surface area, pore size, and isotherm type were further investigated using BET-BJH. The crystal size was calculated using the Scherer equation as follows,

(2) $$S=\frac{0,9\lambda }{\text{Bcos}\theta }$$

With S = Crystal Size (nm)

*λ* = Wavelength (A).
B = FWHM (full width half maximum)(rad)
*θ* = Angle with High Intensity(^o^)

## Ethics Statement

The research does not involve using humans and animals as subjects, and the data were not collected from social media platforms. The samples were taken with verbal consent from the distributors in collaboration with the Muslim Nusantara Alwashliyah University community service.

## CRediT authorship contribution statement

**Khairiah Khairiah:** Data curation, Writing – original draft. **Erna Frida:** Methodology, Investigation. **Kerista Sebayang:** Visualization, Investigation. **Perdinan Sinuhaji:** Visualization, Investigation. **Syahrul Humaidi:** Writing – review & editing.

## Declaration of Competing Interest

The authors declare that they have no known competing financial interests or personal relationships which have or could be perceived to have influenced the work reported in this research.
